# Reduced Dietary Protein and Essential Amino Acids Impair Growth Performance and Increase Lysine Sensitivity in Broiler Chickens

**DOI:** 10.3390/ani15071027

**Published:** 2025-04-02

**Authors:** Paloma Cordero, Galia Ramírez-Toloza, Pablo Dufflocq, Sofía Herrera-Alcaíno, Sergio A. Guzmán-Pino

**Affiliations:** 1 Programa de Doctorado en Ciencias Silvoagropecuarias y Veterinarias, Campus Sur, Universidad de Chile, Santiago 8820808, Chile; paloma.cordero@veterinaria.uchile.cl (P.C.); sofia.herrera.a@ug.uchile.cl (S.H.-A.); 2 Departamento de Fomento de la Producción Animal, Facultad de Ciencias Veterinarias y Pecuarias, Universidad de Chile, Santiago 8820808, Chile; 3 Departamento de Medicina Preventiva Animal, Facultad de Ciencias Veterinarias y Pecuarias, Universidad de Chile, Santiago 8820808, Chile; galiaram@uchile.cl; 4 Food Quality Research Center, Universidad de Chile, Santiago 882080, Chile; prdufflo@ug.uchile.cl

**Keywords:** amino acids, broiler chicken, crude protein, sensory-motivated intake, taste preferences

## Abstract

Nutrition plays a fundamental role in poultry production. Developing high-quality diets that ensure positive feed perception by birds is a key strategy for enhancing production performance within the industry. This study evaluated the effect of crude protein (CP) and four essential amino acid (AA) reductions on broiler chickens’ growth performance and taste sensitivity for Lysine, Methionine, Threonine, and Tryptophan. Sixty-four one-day-old male broiler chickens (Ross 308) were exposed to four dietary treatments consisting of a balanced (control) diet with a total inclusion of CP and Lysine, Methionine, Threonine, and Tryptophan (T1); diets with a 30 g/kg reduction in CP maintaining 100% exogenous incorporation of the four aforementioned AAs (T2); diets with a 30 g/kg reduction in CP and 50% exogenous incorporation of the same four AAs (T3); and diets with a 30 g/kg reduction in CP with no exogenous incorporation of the four previously mentioned AAs (T4). After 7 days of acclimatization to the diets, the birds were offered Lysine, Methionine, Threonine, and Tryptophan at eight concentrations (0.1, 0.5, 1.0, 1.5, 2.0, 2.5, 3.0, and 3.5%) each for 4 h/day in a double-choice test for 32 days to determine the preferences and sensory-motivated intake (SMI) of the birds. During the 39-day trial, the birds in the T4 group showed a lower (*p* < 0.050) average daily feed intake (ADFI), average daily gain (ADG), feed conversion ratio (FCR), and body weight (BW) than the birds of the rest of the treatments. No significant effects of the different dietary treatments, CP content, or AA levels were observed on the preferences or SMI of the birds (*p* > 0.050). However, differences were found in the preferences and SMI between birds from the different treatments when evaluating the treatment × AA × concentration interaction (*p* < 0.050), reflecting that dietary treatments generate changes in the consumption behavior of birds in response to the delivery of AAs at different concentrations. The preference and SMI thresholds for Lysine decreased in the birds in the T4 group, while the thresholds for Methionine and Tryptophan increased compared to the birds in the control group (*p* < 0.050). We concluded that the reduction in CP and AAs in the diet of broiler chickens affects their productive parameters and increases their taste sensitivity, which was reflected in lower weight gain and feed conversion efficiency, along with increased preferences and higher SMI values in the birds exposed to diets with more significant nutritional deficits.

## Simple Summary

This study aimed to evaluate the impact of different diets on growth performance and taste sensitivity in broiler chickens. Specifically, it focused on determining the birds’ preferences and sensory-motivated intake (SMI) for four amino acids (AAs) in diets that were deficient in crude protein (CP) and the same amino acids. The results indicated that reducing CP and AAs in these diets led to decreased feed intake, weight gain, feed conversion, and body weight, in addition to heightened taste sensitivity in the birds, particularly to Lysine, a limiting amino acid. This sensitivity increase significantly improved the birds’ preference and motivation to consume exogenous amino acid compounds. A better understanding of taste sensitivity and its link to feeding behavior in response to external nutritional stimuli in birds is crucial for creating feeding strategies that improve their performance.

## 1. Introduction

Nutrition is essential for achieving optimal productivity in poultry farming. Efforts are currently underway to formulate diets with lower crude protein (CP) levels to enhance economic profitability by directly reducing feed costs [1], promote sustainability by minimizing the release of nitrogenous compounds into the environment [2,3,4], and improve bird health and welfare by mitigating the incidence of certain diseases [5,6,7,8,9]. Thus, numerous investigations have aimed to determine the optimal percentage reduction in dietary CP that maintains bird growth and productivity without compromising performance while remaining suitable for highly intensive commercial systems [4]. A tangible reduction of 20–30 g/kg in dietary CP has been proposed as a conservative approach to minimize negative impacts on broiler performance. Researchers have reported that more significant reductions compromise growth performance and affect meat quality due to increased lipid deposition in the carcass, resulting from disruptions in the birds’ energy balance. Therefore, CP reduction should be carefully implemented under the concept of “ideal protein” to ensure the formulation of nutritionally adequate and well-balanced diets [4].

Changes in dietary formulation can significantly affect the nutritional status of living organisms, influencing their consumption behavior, a factor that has been historically investigated [10,11,12,13,14]. This effect is due to the nutrients in diets functioning as external chemical stimuli that trigger endogenous signals that translate into responses associated with appetite and the control of food intake [15]. This physiological mechanism is conditioned by the functioning of the gut–brain axis, which communicates the nutritional status of the chickens with their central nervous system, generating the activation of enteroendocrine cells after contact with food in the intestinal lumen [15]. This process relies on chemosensors present in the gastrointestinal tract that sense protein-derived compounds from diets such as taste receptor members 1 and 3 (T1R1 and T1R3); the metabotropic glutamate receptors 1 and 4 (mGluR1 and mGluR4); the calcium-sensing receptor (CaSR), the G protein-coupled receptor 92 (GPR92); and the receptors that sense lipid-derived compounds from diets, such as free fatty acid receptors 2, 3, and 4 (FFAR2, FFAR3, and FFAR4) [16,17]. In broiler chickens, these sensors are located along the gastrointestinal tract, from the oral cavity on the palate and base of the tongue, passing through the crop, proventriculus, gizzard, and both anterior (duodenum, jejunum, and ileum) and posterior (cecum and colon) intestines. Their expression levels differ based on the chickens’ age, the intestinal portion and/or tissue, and according to the family of receptors to which it belongs, which is closely related to its functionality and physiology [18].

In this way, through taste sensitivity, birds perceive the nutritional value and dietary deficiencies of foods and adapt their consumption [19]. This capability is vital for maintaining optimal nutritional status [17,20]. The gustatory sensitivity of birds has been tested through preference studies that determine differential consumption of gustatory compounds [21,22,23,24] and the analysis of consumption behaviors in response to such assays [24]. A preference study consists of a test that provides two simultaneous options (diets or solutions), individual birds or groups, followed by an evaluation of compound intake over a designated period [25] so that a greater expression of preference will be reflected in an animal through greater consumption of a particular compound. Preferences have been reported to vary within an organism based on specific factors such as nutritional requirements [11], which has been corroborated in studies of gustatory sensitivity among individuals with different nutritional statuses [12,13,14]. On the other hand, sensory-motivated intake (SMI) refers to a compound’s ability to enhance feeding efficiency by increasing consumption incentives, potentially increasing appetite for a particular compound [19]. This concept involves understanding how birds use their sense of taste to guide their feeding behaviors and dietary choices [17], which is why it has been applied in studies of taste sensitivity in birds [24].

Previous studies of taste preferences in birds for sapid compounds are characterized by a significant degree of variability in their results, which is important given the versatility of the experimental designs [17,21,22,23]. In a recent analysis, we identified essential experimental variables for developing an optimal design for taste preference studies in broiler chickens. Our findings suggest that the most effective model includes early-stage birds tested in pairs per pen, using a liquid matrix for delivering taste compounds [24]. It should be noted that published studies on taste sensitivity and consumption behavior in birds have all offered fully balanced diets, so studies that analyze the type of diet given to birds would be required as a new variable to consider. In contrast, numerous studies have evaluated the effects of reducing CP and AAs in the diet on production parameters and poultry meat quality [4]. However, none have taken a behavioral approach to birds’ voluntary consumption. Therefore, analyzing the impact of these diets on behavioral indicators becomes the main attribute of innovation and originality of the present study.

In this study, we hypothesized that a reduction in CP and four essential amino acids (AAs) affects the growth performance and consumption behavior of broiler chickens, which will be reflected in lower weight gain and feed conversion efficiency as well as changes in sensitivity thresholds and consumption motivation for the same AAs. Therefore, this study aimed to evaluate the effect of CP and four essential AA reductions on broiler chickens’ growth performance and taste sensitivity for Lysine, Methionine, Threonine, and Tryptophan.

## 2. Materials and Methods

### 2.1. Animals and Housing

All the procedures described in this study were conducted at the Experimental Unit for Poultry Nutrition and Production of the Faculty of Veterinary and Animal Sciences (FAVET) of the University of Chile (UCH). Sixty-four one-day-old male broiler chickens (Ross 308) purchased from a commercial company in the Metropolitan Region of Chile were used for a 39-day field trial. The chickens were housed in 32-floor pens within the experimental poultry unit, maintained under a semi-controlled environment. This setup included a conventional shed with manual ventilation using side curtains, gas hoods for temperature regulation, and wood-shaving bedding for improved comfort and hygiene. The 32 pens were randomly assigned to 4 dietary treatments offered ad libitum throughout the experimental period. Water was also offered ad libitum until the start of the tests.

### 2.2. Experimental Diets

The diets used in this study are shown in Table 1. The control treatment (T1) consisted of fully balanced diets that covered all the nutritional requirements of Ross broiler chickens to achieve optimal biological performance [26]. This implied the recommended exogenous incorporation of the unbound synthetic AA Lysine, Methionine, Threonine, and Tryptophan (Veterquímica S.A., Santiago, Chile) into the initial and final diets. Treatment 2 (T2) consisted of initial and final diets formulated under a reduction of 30 g/kg of CP and the total exogenous incorporation of the 4 AAs previously mentioned according to the same inclusion assigned to the control diet. Treatment 3 (T3) consisted of initial and final diets formulated under a reduction of 30 g/kg of CP and the partial exogenous incorporation of the 4 AAs previously mentioned, consisting of 50% of that assigned to T1. Finally, treatment 4 (T4) consisted of initial and final diets formulated under a reduction of 30 g/kg of CP without the exogenous incorporation of the 4 AAs previously mentioned, implying the sole contribution of AAs from the basal ingredients composing the diet. The chemical composition of the four dietary treatments was analyzed according to the Association of Official Analytical Chemists (AOAC) guidelines for the content of moisture (method 945.15), crude protein (Kjeldahl method 945.18, N × 6.25), ether extract (method 945.16), ash (method 920.153), and crude fiber (method 991.36) [27]. The nitrogen-free extract was calculated by difference.

### 2.3. Experimental Design

**Figure 1:**
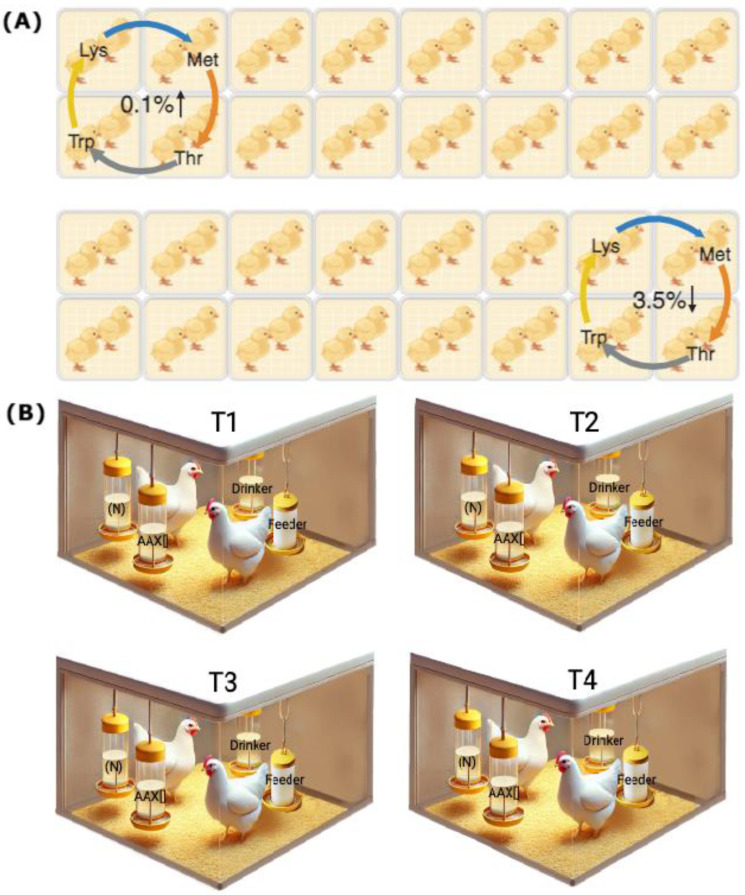
Experimental design diagram. (**A**) Schematic representation of the first testing day illustrates the compound delivery methodology for the double-choice tests. Lysine (Lys), Methionine (Met), Threonine (Thr), and Tryptophan (Trp) were administered to each pen in a counterbalanced manner, covering both the minimum and maximum concentrations. These compounds were rotated over 32 days across the 32 pens of the experimental poultry unit, ensuring that each concentration was tested. (**B**) Schematic representation of the double-choice test setup for assessing the preferences and SMI in four pens, each corresponding to one of the four dietary treatments (T1, T2, T3, and T4). Each test used two liquid matrices containing the neutral compound (N) and the AA solution at a specific concentration (AA × []), with two chickens per pen serving as the experimental unit.

After arriving at the experimental unit, the one-day-old birds were distributed homogeneously according to their body weight in pairs in the floor pens of the experimental unit. They were subjected to 7 days of acclimatization to the environmental conditions before the start of the tests. Throughout the 32-day experimental period, the birds were provided with solutions of Lysine, Methionine, Threonine, and Tryptophan, each diluted in water at eight different concentrations, 0.1, 0.5, 1.0, 1.5, 2.0, 2.5, 3.0, and 3.5%, determining a total of 32 combinations evaluated during 32 days of testing. Each combination was assigned to 8 pens, determining the delivery of each AA in 2 pens, allowing for each compound to be tested in a counterbalanced manner at the minimum and maximum concentrations at the same time [19,29,30], as shown in Figure 1A. Body weight (BW) and feed consumption were registered weekly, allowing for the estimation of the average daily gain (ADG), average daily feed intake (ADFI), and feed conversion ratio (FCR) of birds in each treatment.

The preferences and SMI tests were developed by placing in each pen two identical matrices following the methodology of previous studies [21,22,24,31]. One of them contained drinking water (neutral option, “N”), and the other was one of the four AAs diluted in water at a specific concentration (compound × concentration, “AA × []”; Figure 1B). The tests lasted four hours, starting at 09:00 a.m. and continuing until 1:00 p.m., with a previous fasting hour at 08:00 a.m. During this period, access to the ad libitum delivery drinkers was restricted within each pen. All the AA × [] combinations were tested during the 32 days of assay, and the average consumption of each drinker was estimated by the weight loss of the matrices through the subtraction between the amount delivered and the amount withdrawn. The average daily consumption of the birds obtained on each day of the test was weighted by their metabolic weight to equalize the differential consumption capacities associated with the age of the birds. It was expressed in g/kg of BW. The preference value was calculated as the percentual consumption of AA × [] concerning the total intake (AA × [] consumption + N consumption) and was compared with the neutral value of 50% as previously described [29,30]. The SMI value was calculated as the difference between the consumption of the delivery matrices and compared without consumption (0 consumption). A “+” value indicated a greater consumption of AA × [] offered; in contrast, a “−” value indicated a greater consumption of N. This measurement complements the result of preferences, which is important given that it has been studied that a most preferred compound may not necessarily be the most consumed [19].

### 2.4. Statistical Analysis

The Shapiro–Wilk and Levene’s tests of normality and homogeneity of variance were performed on each variable before statistical analysis. Each dietary treatment’s productive parameters (ADFI, ADG, FCR, and BW) were evaluated using one-way ANOVA and Bonferroni post hoc multiple comparisons. Similarly, the effect of the dietary treatments and their composition factors (CP and AA inclusion levels), as well as the interactions between the dietary treatments and the consumption of compounds at different concentrations offered on the bird’s preferences and SMI tests (treatment × AA × []) were studied using three-way ANOVA and Bonferroni post hoc multiple comparisons. Furthermore, the mean preference and SMI values for each AA × [] analyzed were compared with the neutral value of preference (50%) and without consumption (0) by Student’s *t*-test. For all the analyses, a significance level α of 0.050 was considered. All the analyses and figures were conducted with RStudio software (version 4.1.3, Boston, MA, USA) and GraphPad Prism (version 8.0.2, Boston, MA, USA).

## 3. Results

### 3.1. Feed Intake and Growth Performance

Table 2 shows the results of the broiler chickens’ feed intake and growth performance in response to the dietary treatments. No significant differences were observed in the initial BW of the birds at the beginning of the trial (*p* = 0.480). During the first 7 days, there were no differences in the ADFI of the treated birds and the ADG between groups T1, T2, and T3, but a higher ADG was found in group T1 compared to T4 (*p* = 0.044). During this period, no effect of the treatments on the FCR was observed (*p* = 0.324). Meanwhile, the BW of the birds in treatments T1, T2, and T3 was higher than in treatment T4 (*p* = 0.041). In the second period, between days 8–14 of the trial, no differences were observed between the ADFI of the birds (*p* = 0.661). On the other hand, the effect of the treatments on the ADG and FCR of the T4 group was observed to be lower than the rest of the treatments (*p* < 0.050). Likewise, a significantly higher BW was found in the T1 group compared to the T3 and T4 groups (*p* < 0.0001). During days 15–21 of the trial period, there were also no differences in the ADFI of the birds, but differences were observed in the ADG, FCR, and BW, being lower in the birds of the T4 group compared to the rest of the treatments (*p* < 0.050). Between days 22 and 28 of the trial, differences in ADFI were observed, which was reflected in a higher intake by the birds in the T4 group and a lower intake by the birds in the T2 group (*p* = 0.048). The ADG continued to be significantly higher in the birds in the T1 group compared to the rest of the treatments, while the FCR was significantly lower in the birds in the T4 group than in the T1 group (*p* < 0.050). The BW of the birds in groups T2, T3, and T4 decreased linearly compared to group T1 (*p* < 0.0001). In the last period of the cycle between days 29 and 39, the effect of the treatments on the ADFI, ADG, FCR (*p* < 0.050), and BW (*p* < 0.0001) was observed, which was reflected in the higher values in the birds of group T1 compared to the birds of group T4. A global analysis of the trial that considered days 1 to 39 of the cycle showed significant differences in the ADFI, ADG, FCR, and BW of the birds given the effect of the treatments (*p* < 0.050), which determined that all the parameters were higher in the T1 group compared to the T4 group.

### 3.2. Taste Preferences for Amino Acids

**Figure 2:**
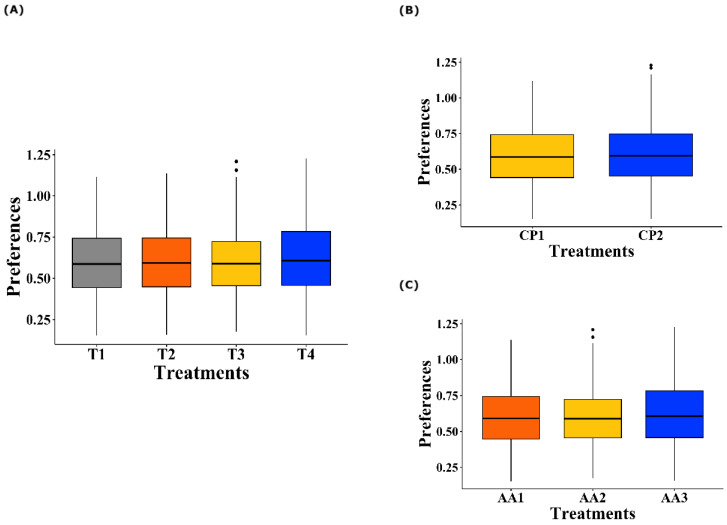
Boxplot of taste preferences of broiler chickens for Lysine, Methionine, Threonine, and Tryptophan. (**A**) Effect of dietary treatments on AA preferences. T1 was the balanced diet with 100% CP and exogenous supplementation of Lysine, Methionine, Threonine, and Tryptophan; T2 was the diet with a 30 g/kg reduction in CP and 100% exogenous four aforementioned AAs; T3 was the diet with a 30 g/kg reduction in CP and 50% exogenous incorporation of the same four AAs; and T4 comprised diets with a 30 g/kg reduction in CP and no exogenous incorporation of the four previously mentioned AAs. (**B**) Effect of the CP inclusion levels on dietary treatments. CP1 indicates 100% CP inclusion, and CP2 represents a 30 g/kg reduction. (**C**) Effect of the AA inclusion levels on dietary treatments. AA1 corresponds to 100% inclusion, AA2 to 50% inclusion, and AA3 to 0% inclusion.

Regarding the treatment effect (Figure 2A), no significant differences were observed in the mean preference values of the birds for all the AAs offered (*p* = 0.730). Similarly, when analyzing the level of CP inclusion in the diet (100% inclusion vs. reduction of 30 g/kg, Figure 2B), no significant effects were observed on the AA preferences of the birds (*p* = 0.527). Finally, when evaluating the different levels of AA inclusion according to the nutritional requirements of the birds (100%, 50%, or 0% inclusion, Figure 2C), no significant differences were detected in the observed preferences (*p* = 0.540).

**Figure 3:**
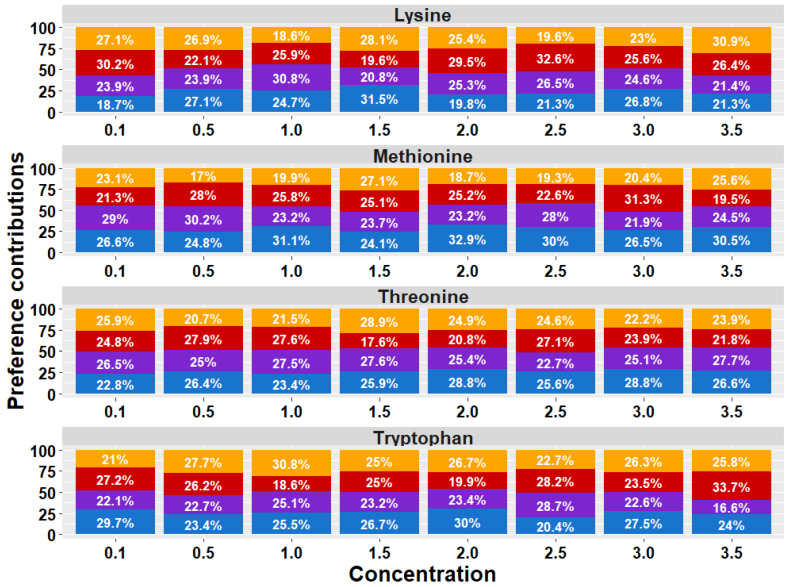
Proportional bar diagram of taste preferences for Lysine, Methionine, Threonine, and Tryptophan at concentrations of 0.1, 0.5, 1.0, 1.5, 2.0, 2.5, 2.0, and 3.5% in broiler chickens exposed to dietary treatments. T1 (orange bars) was the balanced diet with 100% CP and exogenous Lysine, Methionine, Threonine, and Tryptophan supplementation. T2 (red bars) was the diet with a 30 g/kg reduction in CP and 100% exogenous four aforementioned AAs. T3 (purple bars) was the diet with a 30 g/kg reduction in CP and 50% exogenous of the same four AAs. T4 (blue bars) comprised diets with a 30 g/kg reduction in CP and no exogenous incorporation of the four previously mentioned AAs.

The analysis of the treatment × AA × [] interaction (Figure 3) revealed significant differences in the birds’ preference values for AAs at different concentrations (*p* = 0.032). However, the post hoc tests did not identify specific differences between the groups.

**Figure 4:**
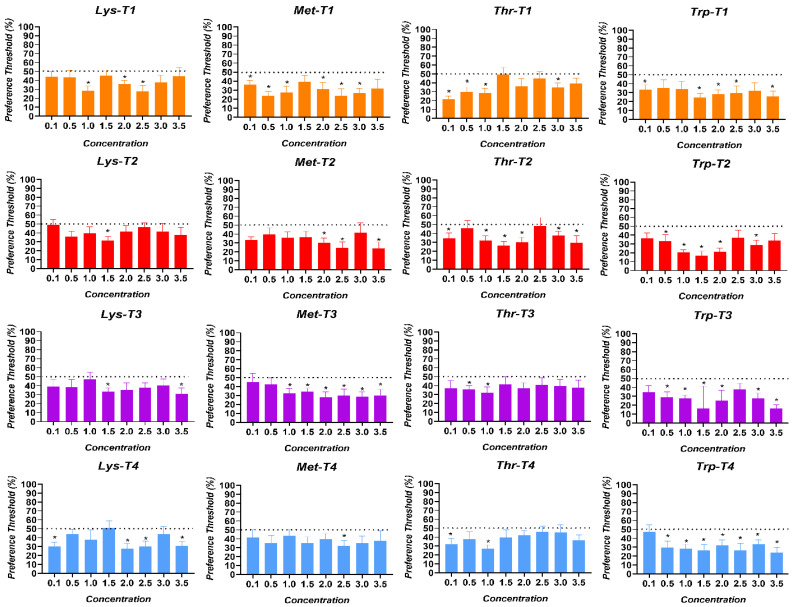
Preference thresholds for Lysine (Lys), Methionine (Met), Threonine (Thr), and Tryptophan (Trp) in broiler chickens exposed to four dietary treatments. T1 was the balanced diet with 100% CP and exogenous supplementation of Lysine, Methionine, Threonine, and Tryptophan. T2 was the diet with a 30 g/kg reduction in CP and 100% exogenous four aforementioned AAs. T3 was the diet with a 30 g/kg reduction in CP and 50% exogenous incorporation of the same four AAs. T4 comprised diets with a 30 g/kg reduction in CP and no exogenous incorporation of the four previously mentioned AAs. The dotted line indicates the neutral or not preference zone (50%). (*) = significant preference values (*p* < 0.050).

The preference thresholds for the AAs in broiler chickens are shown in Figure 4. The AA preference thresholds perceived by the birds in the T1 treatment were at 1.0% for Lysine (*p* = 0.004), 0.1% for Methionine (*p* = 0.021), 0.1% for Threonine (*p* = 0.0005), and at 0.1% for Tryptophan (*p* = 0.023). The birds in the T2 treatment perceived thresholds at 1.5% for Lysine (*p* = 0.005), 0.1% for Methionine (*p* = 0.002), 0.1% for Threonine (*p* = 0.035), and 0.5% for Tryptophan (*p* = 0.048). In turn, the birds exposed to the T3 treatment perceived thresholds at 1.5% for Lysine (*p* = 0.005), 1.0% for Methionine (*p* = 0.012), 0.5% for Threonine (*p* = 0.009), and 0.5% for Tryptophan (*p* = 0.009). Finally, the birds exposed to the T4 treatment perceived thresholds at 0.1% for Lysine (*p* = 0.004), 2.5% for Methionine (*p* = 0.020), 0.1% for Threonine (*p* = 0.026), and 0.5% for Tryptophan (*p* = 0.020).

### 3.3. Sensory-Motivated Intake for Amino Acids

**Figure 5:**
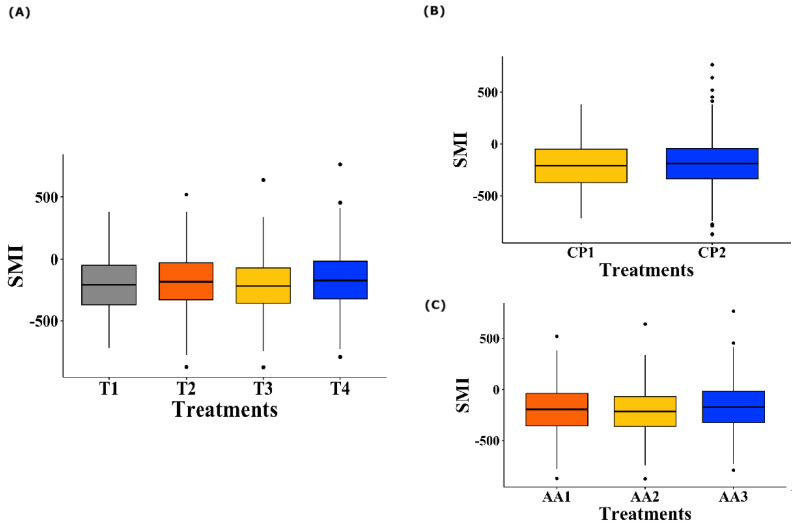
Boxplot of SMI of broiler chickens for Lysine, Methionine, Threonine, and Tryptophan. (**A**) Effect of dietary treatments on AA SMI. T1 was the balanced diet with 100% CP and exogenous supplementation of Lysine, Methionine, Threonine, and Tryptophan; T2 was the diet with a 30 g/kg reduction in CP and 100% exogenous four aforementioned AAs; T3 was the diet with a 30 g/kg reduction in CP and 50% exogenous incorporation of the same four AAs; and T4 comprised diets with a 30 g/kg reduction in CP and no exogenous incorporation of the four previously mentioned AAs. (**B**) Effect of the CP inclusion levels on dietary treatments. CP1 indicates 100% CP inclusion, and CP2 represents a 30 g/kg reduction. (**C**) Effect of the AA inclusion levels on dietary treatments. AA1 corresponds to 100% inclusion, AA2 to 50% inclusion, and AA3 to 0% inclusion.

Regarding the treatment effect (Figure 5A), no significant differences were observed in the SMI values of the birds for all the AAs offered (*p* = 0.081). Similarly, when analyzing the level of CP inclusion in the diet (100% inclusion vs. reduction of 30 g/kg, Figure 5B), no significant effects were observed on the SMI of the birds (*p* = 0.309). Finally, when evaluating the different levels of AA inclusion according to the nutritional requirements of the birds (100%, 50%, or 0% inclusion, Figure 5C), no significant differences were detected in the observed SMI (*p* = 0.100).

**Figure 6:**
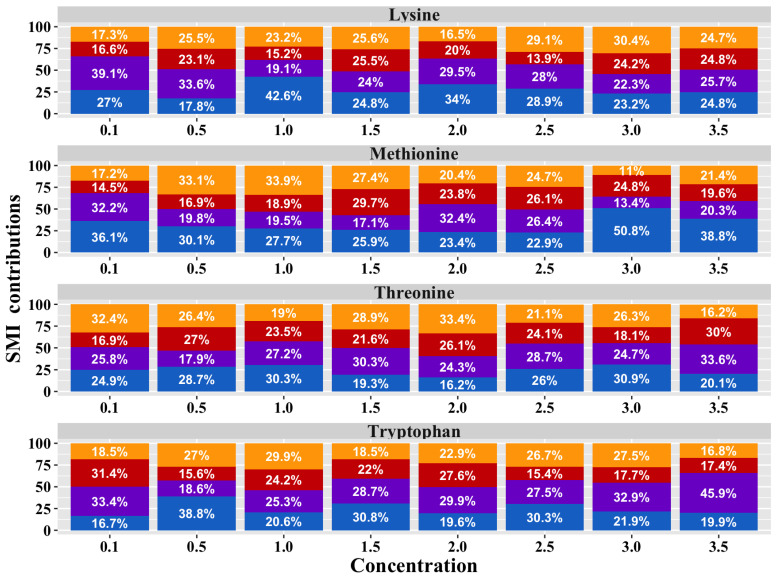
Proportional bar diagram of SMI for Lysine, Methionine, Threonine, and Tryptophan at concentrations of 0.1, 0.5, 1.0, 1.5, 2.0, 2.5, 2.0, and 3.5% in broiler chickens exposed to dietary treatments. T1 (orange bars) was the balanced diet with 100% CP and exogenous Lysine, Methionine, Threonine, and Tryptophan supplementation. T2 (red bars) was the diet with a 30 g/kg reduction in CP and 100% exogenous four aforementioned AAs. T3 (purple bars) was the diet with a 30 g/kg reduction in CP and 50% exogenous incorporation of the same four AAs. T4 (blue bars) comprised diets with a 30 g/kg reduction in CP and no exogenous incorporation of the four previously mentioned AAs.

Similar to that observed in the preference results, the analysis of the treatment × AA × [] interaction (Figure 6) revealed significant differences in the birds’ SMI values for AAs at different concentrations (*p* = 0.001). However, the post hoc tests did not identify specific differences between the groups.

**Figure 7:**
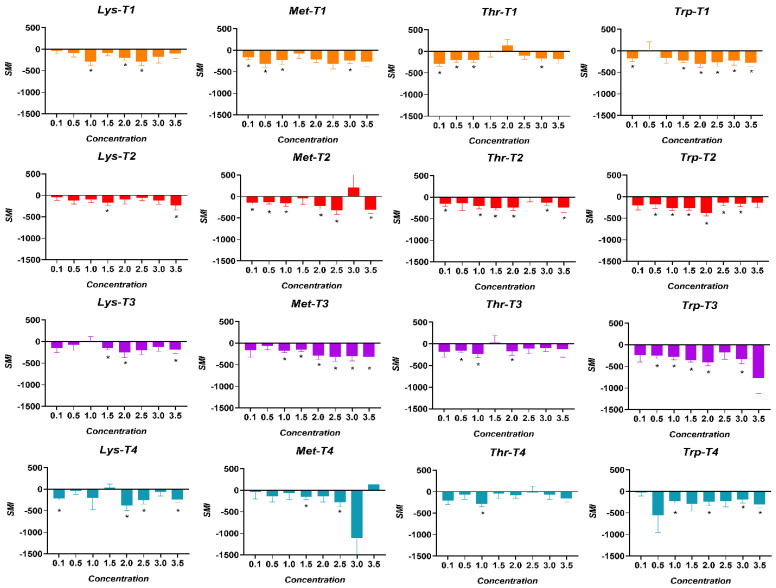
SMI thresholds for Lysine (Lys), Methionine (Met), Threonine (Thr), and Tryptophan (Trp) in broiler chickens exposed to four dietary treatments. T1 was the balanced diet with 100% CP and exogenous supplementation of Lysine, Methionine, Threonine, and Tryptophan. T2 was the diet with a 30 g/kg reduction in CP and 100% exogenous four aforementioned AAs. T3 was the diet with a 30 g/kg reduction in CP and 50% exogenous incorporation of the same four AAs. T4 comprised diets with a 30 g/kg reduction in CP and no exogenous incorporation of the four previously mentioned AAs. (*) = consumption values significantly higher than the negative control (*p* < 0.050).

The SMI thresholds for AAs in broiler chickens are shown in Figure 7. The SMI thresholds of the AAs perceived by the birds of the T1 treatment were for Lysine at 1.0% (*p* = 0.003), for Methionine at 0.1% (*p* = 0.007), for Threonine at 0.1% (*p* = 0.001), and for Tryptophan at 0.1% (*p* = 0.008). The birds in the T2 treatment showed a higher SMI threshold for Lysine at 1.5% (*p* = 0.006), at 0.1% for Methionine (*p* = 0.0001), at 0.1% for Threonine (*p* = 0.005), and 0.5% for Tryptophan (*p* = 0.042). In turn, the SMI thresholds of the birds exposed to the T3 treatment were at 1.5% for Lysine (*p* = 0.001), 1.0% for Methionine (*p* = 0.001), 0.5% for Threonine (*p* = 0.001), and at 0.5% at Tryptophan (*p* = 0.002). Finally, the birds exposed to the T4 treatment showed SMI thresholds at 0.1% for Lysine (*p* = 0.0005), 1.5% for Methionine (*p* = 0.026), 1.0% for Threonine (*p* = 0.034), and at 1.0% for Tryptophan (*p* = 0.001).

## 4. Discussion

The relationship between nutritional status and birds’ feeding behavior is grounded in taste perception’s critical role in nutrient detection and food selection in broiler chickens [32]. This ability enables rapid adaptation in their feeding behavior [19]. The present study investigated the nutritional effects of low protein/amino acid diets on feeding behavior, focusing on the gustatory sensitivity of broiler chickens to varying concentrations of Lysine, Methionine, Threonine, and Tryptophan. Changes in taste sensitivity of AAs not only influence feeding behavior but also impact metabolism, productivity, and bird welfare, as has been observed in the case of Tryptophan [33]. For this study, the birds were divided into four groups, each fed diets with varying levels of unbound essential CP components and AAs. Precisely, three of the four diets were adjusted to include a moderate reduction of 30 g/kg of CP, a level considered to have no negative impact on bird performance [4,34]. Regarding the inclusion of exogenous AAs, diets were designed to include 100%, 50%, and 0% of the balanced amount of each AA required for broiler chickens of the specific genetic line under study. These diets served as treatments to induce differential nutritional statuses among the birds.

Feed intake and growth performance were measured to assess the effects of reduced CP and AA levels on the birds’ productivity and to confirm the nutritional impacts induced when subsequent two-choice preference and SMI tests with AAs were performed. As expected, productive results in this study showed a negative effect of dietary treatments on the ADG and BW of broilers from the first week and on the FCR from the second week of the production cycle onwards, reflected in a reduction in weight gain and feed conversion in birds exposed to restricted dietary treatments. Previous research has indicated that a moderate reduction in CP does not affect the productive performance of birds, provided that these formulations are combined with the strategic inclusion of synthetic essential AAs in sufficient quantities to meet the minimum nutritional requirements that ensure animal productivity [4]. The present study evaluated the use of diets with reduced CP and different levels of inclusion of exogenous AAs, resulting in different degrees of deterioration in the productive performance of birds associated with the type of diet consumed. Birds fed the reduced CP diet with the complete inclusion of essential AAs (T2) did not reflect negative changes in their parameters during the first 2 weeks of the trial, reflecting compensation for the imposed nutritional deficit, thus allowing them to exhibit improved productive responses compared to treatments with CP and AA reductions (T3 and T4) that showed negative productive responses as anticipated based on previously published evidence. It is important to emphasize that the primary goal of reducing dietary CP by adjusting the inclusion of unbound synthetic AAs is to enhance the profitability of poultry production systems by lowering feed costs [35,36]. Equally important, formulating diets with reduced CP and AA levels contributes to minimizing environmental impact under precision nutrition, a key factor in ensuring sustainability in modern animal production [2,3,4,37].

Regarding the results of the preferences and SMI analysis obtained, the hypothesis of this research that diets with reduced CP and four essential AAs induce changes in the feeding behavior of chickens was confirmed. As seen in the proportional bar diagram, an increase in preferences (T2 = 804.5%, T3 = 792.8%, and T4 = 833.2%) and SMI (T3 = 857.1% and T4 = 873.8%) for all the compounds evaluated in the birds was quantified as nutritional restriction increased within the treatments provided, compared to the control treatments (T1 preference = 769.3%; T1 SMI = 772.9%). This result is consistent with previous research that indicates that the quality and quantity of food consumed alters the taste sensitivity in living beings, which is explained because said sensitivity responds to the hormones of hunger and satiety in the body, such as the case of leptin [38]. It has been reported that birds use taste to detect nutritional deficiencies and adjust their feeding behavior to achieve perfectly balanced diets [39]. Oral taste receptors that provide gustatory sensitivity to birds have also been detected throughout the gastrointestinal tract. This expression generates a connection associated with the need to monitor the availability and absorption of nutrients beyond the oral cavity. It responds to the food contained in the lumen and triggers the secretion of intestinal hormones/peptides, such as glucagon-like peptide 1 (GLP1), cholecystokinin that affects appetite and satiety (CCK), or ghrelin. These become signaling molecules that transmit information about nutritional status to the brain, modulating physiological responses to control food consumption [39]. A precedent has been found in mammalian species, such as rats and pigs, on how diet determines taste sensitivity. In young rats, it was reported that consumption behavior and taste sensitivity are influenced by their nutritional status at early stages [40,41]. Similarly, Guzmán-Pino et al. (2014) determined that pigs fed low-CP diets, conditioned by post-ingestive consequences, showed a pattern of selection and preference for flavors associated with protein compounds in an appropriate manner to overcome their state of protein deficiency based on associative learning [42]. In humans, obese individuals have been shown to experience impaired taste sensitivity for sweet and fatty taste compounds compared to thin individuals [12,13,14].

In the same way as protein, it has also been shown that energy requirements condition food preferences, determining the type of sugar individuals choose [11]. It is important to highlight that the key role of taste in food selection and possibly in motivating feeding behavior has been established [17,20]. Increased taste sensitivity to Lysine was detected in birds, leading to an increased preference and SMI for the compound at a lower concentration in bird-fed diets with more significant reductions (T3 and T4). These findings are supported by previous research showing that organisms’ nutritional needs influence their food selection and intake [11].

The preference thresholds were all detected below the 50% preference or neutral zone, meaning the birds in all the treatments showed indifference and/or aversive responses to the compounds offered during the trial. However, it is remarkable that the main finding was the decrease in the Lysine detection threshold in response to the nutritional deficit in the chickens. The SMI thresholds for the AA compounds were also lower than those observed for the neutral compound offered during the trial. This indicates a greater incentive for the birds to consume the neutral compound than the AA offered. Nevertheless, the increased SMI reported for Lysine at a lower concentration supports the preference threshold results and reinforces the experimental hypothesis. An interesting result to discuss is the response observed in birds exposed to the treatments with a more significant protein/amino acid deficit (T3 and T4). They showed higher preferences and SMI thresholds for Methionine and Threonine at higher concentrations compared to the control group, which could potentially be explained as an attempt to compensate for the nutritional deficit due to the imbalance of AAs in the diet [43], this being an important concept to address for future research. Nutritional homeostasis, and particularly energy balance, is recognized as the main driving factor for food intake [44,45], supported by the premise that nutrients in the intestinal lumen can activate cells that secrete peptides, facilitating communication along the gut–brain axis. This pathway reports the nutritional status of birds, triggering physiological responses associated with appetite and intake control [15]. Analyzing birds’ taste perception of dietary nutrients by assessing their sensitivity presents an opportunity to enhance feed formulations [36]. This approach enables the precise inclusion of specific nutrient concentrations to optimize feed intake stimulation, ultimately improving productive performance.

## 5. Conclusions

The dietary reduction in CP and four essential AAs negatively affects broiler chickens’ feed intake and growth performance, increasing their taste sensitivity for Lysine. This study provides important guidelines for adjusting the CP levels and adding exogenous essential AAs to broiler diets. These findings also emphasize the strategic selection of nutritional ingredients to create diets that improve birds’ taste sensitivity and encourage higher feed intake. Poultry diet formulation should prioritize adjustments in CP levels and consider taste sensitivity as a key factor for optimizing nutrition and production efficiency. Further research is needed on birds’ taste sensitivity to various dietary compounds to refine diet formulations and improve nutrition and productivity in poultry systems.

## Figures and Tables

**Table 1:** Composition and chemical analysis of the starter and grower diets of each dietary treatment used in the experiment.

Treatment	T1		T2		T3		T4	
Item	Starter	Grower	Starter	Grower	Starter	Grower	Starter	Grower
Ingredients (g/kg)								
Corn	462.2	470.6	568.7	470.6	598.4	470.6	554.5	665.1
Soybean meal 50%	165.9	39.7	118.3	-	95.1	-	167.3	65.6
Meat meal 60%	170.1	167.7	149.9	126.5	170.7	125.2	107.1	99.5
Ground wheat	-	93	-	171.8	-	180.5	-	-
NaHCO_3_	-	-	-	1.6	-	1.7	1.9	0.3
CaCO_3_	9.7	7.8	1.03	7.8	9.9	7.8	9.8	7.9
NaCl	-	-	-	0.1	0.1	0.1	0.6	0.7
CaHPO_4_	20.2	14.1	22.1	14.2	22.9	14.3	21.4	16.3
Lipofeed 30 AL ^1^	166.4	202.7	121.4	199.7	97.7	195.9	136.5	14.46
Lys	1.44	1.19	1.44	1.19	0.72	0.59	-	-
Met	0.53	0.48	0.53	0.48	0.26	0.24	-	-
Thr	0.97	0.81	0.97	0.81	0.48	0.40	-	-
Trp	0.23	0.19	0.23	0.19	0.11	0.09	-	-
Copitox MB ^2^	0.5	0.5	0.5	0.5	0.5	0.5	0.5	0.5
Multivitamins-mineral-phytase ^3^	2.0	2.0	2.0	2.0	2.0	2.0	2.0	2.0
Analyzed nutrient composition (%)								
Dry matter	88.1	88.6	87.1	88.4	87.5	87.8	87.2	86.2
Crude protein	24.6	21.7	20.3	17.4	20.4	16.7	20.6	15.8
Crude fiber	3.8	4.7	2.9	5.6	3.5	5.1	3.8	3.4
Ether extract	8.7	10.6	7.3	10.1	8.0	9.4	7.8	7.0
Nitrogen-free extract	42.2	43.2	48.4	47.1	46.8	49.1	46.6	54.3
Ash	8.8	8.4	8.2	8.2	8.8	7.5	8.4	5.7
Estimated amino acid content (g/kg) ^4^								
Lys	6.42	4.50	5.45	3.42	4.69	2.80	4.06	2.47
Met	1.88	1.53	1.64	1.27	1.36	1.02	1.10	0.66
Thr	4.06	2.86	3.42	2.25	2.90	1.71	2.50	1.53
Trp	0.85	0.67	0.92	0.37	0.48	0.27	1.50	0.24

^1^ Corn germ oiled with fatty acids vegetable certified and Lecithinated (min 30–40 g/kg of functional soy Lecithin). ^2^ Contains the following per kilo of premix: 10.000 mg of Butylhydroxytoluene, 100.000 mg of Calcium Propionate, 439.796 mg Kieselgur, 200.000 mg of Sepiolites and associated clays, 150.000 mg of Bentonite, and 100.000 mg of *Saccharomyces cerevisiae*. ^3^ Contains the following per kilo of premix: 8000 UI of Vit. A, 3.300 UI of Vit. D3, 16.500 mg of Vit. E, 2.100 mg of Vit. K3, 1500 mg of Vit. B, 15.500 mg of Vit. B2, 3000 mg of Vit. B6, 23 mg of Vit. B12, 8000 mg of Pantothenic Acid, 20.000 mg of Niacin, 50 mg of Biotin, 600 mg of Folic Acid, 30.000 mg of Fe, 80.000 mg of Zn, 7000 mg of Cu, 85.000 mg of Mn, 1000 mg of I, 220 mg of Se, 600 mg of Propil Gaiat, 5000 mg of Butylhydroxytoluene, 600 mg of Beta-apo-8′-carotenal, 6.500 mg of Canthaxanthin, 125.000 OTU of 6-Fitase, 587.500 U of Endo-1,3(4)-beta-glucanase, 4.500.000 U of Endo-1,4-beta-glucanase, 100.000 U of Alpha-amylase, 425.000 U of Bacillolysin, 8.749.999 U of Endo-1,4-beta-xylanase, 1.400 mg of Citric Acid, and 38.663 mg of Saliolitic Clay. ^4^ Calculated values according to Rostagno et al. [28].

**Table 2:** Feed intake and growth performance of broiler chickens exposed to four dietary treatments during a 39-day production cycle ^1^.

Item	T1	T2	T3	T4	SEM	*p*-Value
Days 1 to 7						
Initial BW ^2^	52.5	54.3	53.8	54.6	1.45	0.480
ADFI ^3^	28.6	26.2	29.3	15.2	6.34	0.208
ADG ^4^	8.57 ^a^	7.62 ^a^	7.05 ^ab^	5.33 ^b^	0.793	0.044
FCR ^5^	3.34	3.47	4.19	3.25	0.854	0.324
Final BW	121 ^a^	115 ^a^	110 ^a^	97.2 ^b^	4.62	0.041
Days 8 to 14						
ADFI	31.8	31.4	32.7	30.7	4.56	0.661
ADG	26.9 ^a^	23.1 ^a^	21.7 ^a^	15.3 ^b^	1.910	0.014
FCR	1.18 ^b^	1.36 ^b^	1.52 ^ab^	2.06 ^a^	0.2015	0.010
Final BW	309 ^a^	277 ^ab^	262 ^b^	204 ^c^	12.69	<0.0001
Days 15 to 21						
ADFI	70.8	69.7	71.1	73	4.261	0.443
ADG	55.5 ^a^	49.2 ^ab^	43.2 ^b^	30 ^c^	3.755	0.017
FCR	1.29 ^b^	1.44 ^b^	1.68 ^b^	2.54 ^a^	0.1847	0.0004
Final BW	698 ^a^	621 ^b^	565 ^b^	415 ^c^	26.48	<0.0001
Days 22 to 28						
ADFI	86.7 ^ab^	67.7 ^b^	82 ^ab^	101 ^a^	11.536	0.048
ADG	63.53 ^a^	45.8 ^b^	46.8 ^b^	46.9 ^b^	5.545	0.033
FCR	1.39 ^b^	1.59 ^ab^	1.79 ^ab^	2.46 ^a^	0.450	0.024
Final BW	1142 ^a^	941 ^b^	892 ^b^	743 ^c^	39.69	<0.0001
Days 29 to 35						
ADFI	150 ^a^	115 ^b^	128 ^ab^	116 ^b^	8.695	0.003
ADG	92.6 ^a^	55.1 ^b^	55.5 ^b^	51.3 ^b^	5.705	<0.0001
FCR	1.65 ^b^	2.14 ^b^	2.37 ^a^	2.36 ^a^	0.249	0.049
Final BW	1790 ^a^	1327 ^b^	1280 ^b^	1102 ^c^	55.06	<0.0001
Days 35 to 39						
ADFI	156 ^a^	126 ^b^	143 ^ab^	149 ^ab^	9.220	0.030
ADG	30.8 ^a^	37 ^a^	32 ^a^	19 ^b^	3.574	0.019
FCR	5.19 ^b^	3.62 ^b^	4.78 ^b^	7.96 ^a^	0.7066	0.003
Final BW	1891 ^a^	1482 ^b^	1388 ^b^	1100 ^c^	78.39	<0.0001
Days 1 to 39						
Initial BW	52.5	54.3	53.8	54.6	1.45	0.480
ADFI	81.3 ^a^	67.5 ^b^	75.1 ^a^	74 ^ab^	2.629	0.043
ADG	46.4 ^a^	35.5 ^b^	33.9 ^b^	28.1 ^c^	1.475	<0.0001
FCR	1.76 ^c^	1.90 ^bc^	2.24 ^b^	2.69 ^a^	0.1277	0.007
Final BW	1891 ^a^	1482 ^b^	1388 ^b^	1100 ^c^	78.39	<0.0001

^a–c^ Means in the same row with different superscripts differ (*p* < 0.050). ^1^ Treatment 1 (T1, control) consisted of balanced diets with 100% CP and exogenous supplementation of Lysine, Methionine, Threonine, and Tryptophan, following the Ross broiler chicken nutritional guidelines [26] (Aviagen, 2022). Treatment 2 (T2) involved diets with a 30 g/kg reduction in CP, maintaining 100% exogenous incorporation of the four aforementioned AAs. Treatment 3 (T3) included diets with a 30 g/kg reduction in CP and 50% exogenous incorporation of the same four AAs. Lastly, treatment 4 (T4) comprised diets with a 30 g/kg reduction in CP and no exogenous incorporation of the four previously mentioned AAs. ^2^ Body weight. ^3^ Average daily feed intake. ^4^ Average daily gain. ^5^ Feed conversion ratio.

## Data Availability

The data are available upon reasonable request to the submitting author.
